# Sodium valproate stimulates potassium and chloride urinary excretion in rats: gender differences

**DOI:** 10.1186/1471-2210-7-9

**Published:** 2007-08-06

**Authors:** Eitautė Jakutiene, Jurgita Grikiniene, Arunas Vaitkevicius, Marina Tschaika, Janina Didziapetriene, Donatas Stakisaitis

**Affiliations:** 1 Vilnius University Children's Hospital, Vilnius, Lithuania; 2 Neurology and Neurosurgery Clinic, Vilnius University, Vilnius, Lithuania; 3 PharmaEast GmbH, Berlin, Germany; 4 Institute of Oncology, Vilnius University, Vilnius, Lithuania

## Abstract

**Background:**

The diuretic effect of valproates and its relation to urinary potassium (K^+^) and chloride (Cl^-^) excretion have not yet been investigated, so the aim of this study was to evaluate the influence of a single dose of sodium valproate (NaVPA) on 24-h urinary K^+ ^and Cl^- ^excretion in young adult Wistar rats of both genders. For measurement of K^+ ^in urine, the same animals and samples as in our earlier publication were used (Pharmacology 2005 Nov, 75:111–115). The authors propose a new approach to the pathophysiological mechanisms of NaVPA effect on K^+ ^and Cl^- ^metabolism.

Twenty six Wistar rats were examined after a single intragastric administration of 300 mg/kg NaVPA (13 NaVPA-male and 13 NaVPA-female), 28 control intact Wistar rats (14 males and 14 females) were studied as a control group. The 24-h urinary K^+^, Cl^-^, creatinine and pH levels were measured.

**Results:**

Total 24-h diuresis and 24-h diuresis per 100 g of body weight were found to be significantly higher in NaVPA-rats of both genders than in rats of the control group (p < 0.05). The data showed NaVPA to enhance 24-h K^+ ^excretion in NaVPA-males and NaVPA-females with significant gender-related differences: 24-h K^+ ^excretion in NaVPA-male rats was significantly higher than in control males (p = 0.003) and NaVPA-female rats (p < 0.001). Regarding the 24-h K^+ ^excretion, NaVPA-female rats did not show a statistically significant difference versus females of the control group (p > 0.05). 24-h urinary K^+ ^excretion per 100 g of body weight in NaVPA-male rats was significantly higher than in control males (p = 0.025). NaVPA enhanced Cl^- ^urinary excretion: 24-h Cl^- ^urinary excretion, 24-h urinary Cl^- ^excretion per 100 g of body weight and the Cl^-^/creatinine ratio were significantly higher in NaVPA-male and NaVPA-female rats than in gender-matched controls (p < 0.05). 24-h chloriduretic response to NaVPA in male rats was significantly higher than in female rats (p < 0.05).

**Conclusion:**

NaVPA causes kaliuretic and chloriduretic effects with gender-related differences in rats. Further investigations are necessary to elucidate the mechanism of such pharmacological effects of NaVPA.

## Background

Currently there are experimental data that valproate (branched-chain fatty acid, valproic acid) increases the turnover of γ-aminobutyric acid (GABA) and thereby potentiates GABAergic functions [1]. The specificity of valproate for GABA suggests that this interaction may be an important mechanism through which sodium valproate (NaVPA) exerts its pharmacological effects [2]. Recently NaVPA has shown to enhance the urinary excretion of sodium (Na^+^) and chloride (Cl^-^) ions in both genders, but the 24-h chloriduretic response in male rats to NaVPA was significantly higher than in female rats [3]. The effect of NaVPA on potassium ion (K^+^) excretion was not yet studied.

The aim of the present study was to evaluate the effect of NaVPA on urininary K^+ ^and Cl^- ^excretion in Wistar rats of both genders and to discuss the NaVPA effects on K^+ ^and Cl^- ^metabolism that could be related to NaVPA pharmacological properties.

The GABA type A receptor (GABA(A)) is an ionotropic receptor. Its subunits form a functional Cl^- ^channel [4,5]. The GABA(A) receptor subunits are expressed in Wistar rat kidney proximal convoluted and straight tubules [6]. The GABA(A) receptor is rapidly activated by valproate in the brain [7]. Cl^- ^channels play a critical role in the functioning of the nervous system by asserting control over voltage potentials across the plasma membrane [8]. There are gender-related differences in Cl^- ^transport across the cell membrane, intracellular Cl^- ^level and the sensitivity of Cl^- ^transport to vasopressin in smooth muscle cells of rats [9]. Intracellular Cl^- ^level and Cl^- ^transport differences could be important in the regulation of cellular processes and could help to explain certain functional differences of cells [9,10]. Cl^- ^is an important factor of intracellular pH [11], which is involved in the complex of cell function regulation.

Investigations show that K^+^-Cl^- ^cotransport takes part in the regulation of signaling pathways involved in several tissue and cell types from different species [12]. In modeling Cl^- ^transport in the rat proximal tubule, Weinstein has found that Cl^- ^ions efflux from the cell predominantly via the K^+^-Cl^- ^cotransport mechanism [13]. The intracellular Cl^- ^level is dependent upon the K^+^-Cl^-^co-transporter (KCC) that determines whether neurons respond to GABA by depolarization or hyperpolarization. However, the role of KCC-dependent chloride homeostasis in the regulation of spontaneous activity of neuronal circuits via GABA(A) receptor is still unknown. Findings suggest that KCC-dependent chloride homeostasis is mainly involved in GABA(A) receptor-mediated synaptic inhibition [14]. There are no investigational data on the interaction between KCC and GABA receptors, K^+ ^homeostasis or NaVPA effects on K^+ ^and Cl^- ^transport in the kidney.

The study provides data to show that NaVPA in rats, along with the known diuretic and chloriduretic effects, causes also a kaliuretic effect that has not yet been investigated. For measurement of K^+ ^in urine, the same animals and samples as in our earlier publication were used (Pharmacology 2005 Nov, 75:111–115).

## Methods

Twenty-six Wistar rats (13 NaVPA-males and 13 NaVPA-females) were examined after a single intragastric dose of 300 mg/kg sodium valproate (Convulex, 300 mg/ml, drops (water solution, pH 9.0), Gerot Pharmazeutika Wien, Austria). In addition, 28 Wistar intact rats (14 males and 14 females) were examined as a control group. NaVPA dosage was chosen in accordance with data of preclinical pharmacodynamic studies of NaVPA [15]. The study was approved by the Lithuanian Committee for Animals Care and Use (No. 0019; 2005). The mean age of control rats was 91 ± 9 days for males and 90 ± 8 days for females, and the mean age of NaVPA-rats was 97 ± 10 days for males and 95 ± 9 days for females. The mean weight of male rats was 283 ± 30 g in control group and 298 ± 23 g in NaVPA-rats. The mean weight of female rats was 236 ± 18 g in control and 240 ± 16 g in NaVPA-rats. The weight was significantly higher in male than in female rats in both groups (p < 0.05).

The animals were housed in standard colony cages with free access to food (chow pellets) and tap water. The room temperature was 21 ± 1°C. The rats were on a natural light-dark cycle. All experiments were performed according to the institutional guidelines for animal care in order to avoid any unnecessary distress to the animals and to reduce the number of animals used. The animals were housed in described conditions and acclimated for at least 5 days before experiments. 24-h urine was collected keeping a rat alone in a special cage (diuresis cage for rats 3700D000/3701D000, Tecniplast, Italy) for 24 h (from 9:00 a.m. till 9:00 a.m. of the next day) with free access to tap water, without food, in the same temperature and light conditions. 24-h urine was collected after a single dose administration.

24-h urinary K^+^, Cl^- ^levels were analyzed with an EML-105 electrolyte analyzer (Radiometer, Denmark). Urinary pH levels were measured with a pH/mV/ion meter (ION Meter pH 340/ION, Germany).

We calculated the 24-h excretion of K^+^, Cl^-^, creatinine, K^+^/creatinine, Cl^-^/creatinine ratio, as well as 24-h diuresis and 24-h urinary K^+^, Cl^- ^excretion per 100 g of body weight. Data were expressed as mean ± SD values from *n *animals. Comparisons between the groups were carried out using Student' s t test. A value of p < 0.05 was considered significant. Correlations between two variables were investigated by the method of linear correlation analysis. The Pearson correlation coefficient r, which represents the linear relationship between two variables, was applied; a value of p < 0.05 was considered significant. STATISTICA for Windows software (StatSoft, USA, 1995) was used to perform the analysis of our data.

## Results

### Diuresis in control and NaVPA-rats

24-h diuresis in control rats showed no statistically significant gender-related differences (p > 0.05). 24-h diuresis per 100 g of body weight in control female rats (3.90 ± 1.10 ml/100 g) was significantly higher (p < 0.02) than in control male rats (2.89 ± 0.91 ml/100 g). After a single intragastric administration of 300 mg/kg NaVPA, 24-h diuresis was significantly (p < 0.05) higher in both genders as compared to control groups, without statistically significant gender-related differences (Table 1). 24-h diuresis per 100 g of body weight in NaVPA-females (5.04 ± 1.66 ml/100 g) and NaVPA-males (5.38 ± 2.41 ml/100 g) was significantly higher (p < 0.05) than in control female and male rat groups (3.90 ± 1.10 ml/100 g, 2.89 ± 0.91 ml/100 g, respectively), with no statistically significant gender-related differences.

**Table 1:** Diuresis and 24-h urinary K^+ ^excretion in male and female control and NaVPA-rat groups (mean ± SD)

Rat groups	n	24-h diuresis (ml)	24-h K^+ ^level (mmol/l)	24-h K^+ ^excretion (mmol)	24-h K^+ ^excretion per 100 g body weight (mmol)	K^+^/creatinine ratio
Control rats						
females	14	9.1 ± 2.3	116 ± 27	1.01 ± 0.17	0.43 ± 0.06*	13.9 ± 3.38
males	14	8.0 ± 2.1	135 ± 29	1.04 ± 0.18	0.37 ± 0.08*	12.7 ± 7.9
NaVPA-rats						
females	13	12.1 ± 4.1^•^	84 ± 28^•^	0.93 ± 0.14*	0.39 ± 0.06	11.8 ± 2.34
males	13	16.0 ± 7.2^•^	93 ± 30^•^	1.32 ± 0.26^• ^*	0.44 ± 0.08^•^	12.4 ± 2.88

^• ^– Statistically significant difference as compared to control group (p < 0.05).

* – Statistically significant difference versus the other gender (p < 0.05).

**Figure 1:**
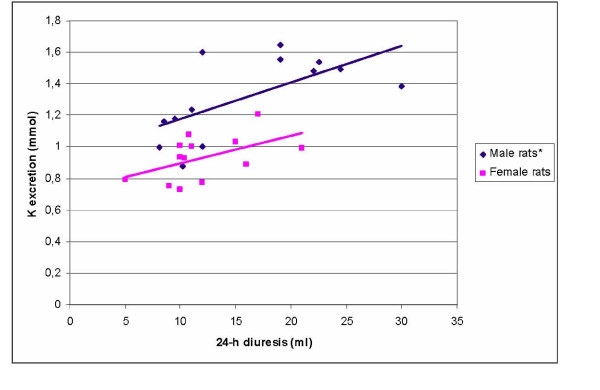
Correlation between 24-h diuresis and K^+ ^excretion in NaVPA male and female rats. * – Statistically significant correlation.

**Figure 2:**
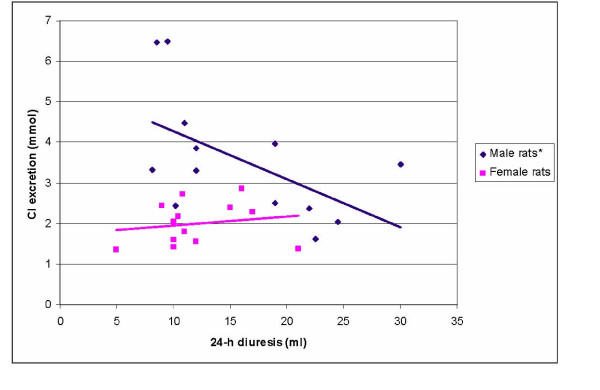
Correlation between 24-h diuresis and Cl^- ^excretion in NaVPA male and female rats. * – Statistically significant correlation.

**Figure 3:**
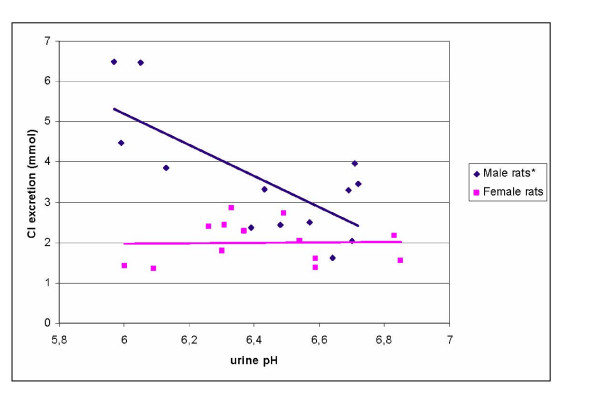
Correlation between urine pH and Cl^- ^excretion in NaVPA male and female rats. * – Statistically significant correlation.

No statistically significant correlation between 24-h diuresis and 24-h urinary K^+ ^excretion or between 24-h diuresis and 24-h urinary Cl^- ^excretion was found in male and female control rats (p > 0.05). Statistically significant correlations between 24-h diuresis and 24-h K^+ ^excretion (r = 0.64; p < 0.05) were found in NaVPA-males. There were no statistically significant correlations between diuresis and 24-h urinary K^+ ^excretion in NaVPA-females (p > 0.05, Figure 1). In NaVPA-male rats, the negative correlation between Cl^- ^excretion and 24-h diuresis (r = -0.56) and between Cl^- ^excretion and urine pH (r = -0.73) was significant (r = -0.73; p < 0.05), and no such correlations were found for NaVPA-female rats (r = 0.18 and r = 0.02; p > 0.05, Figure 2, 3).

### K^+ ^excretion in control and NaVPA-rats

No statistically significant gender-related differences in 24-h urine K^+ ^excretion and in the K^+^/creatinine ratio were determined in control rats (p > 0.05). 24-h urinary K^+ ^excretion per 100 g of body weight was significantly higher in control females than in control males (p = 0.04; Table 1). 24-h K^+ ^excretion in NaVPA-male rats was significantly higher than in control male (p = 0.003) and NaVPA-female rats (p < 0.001). 24-h K^+ ^excretion in NaVPA-female rats did not show statistically significant difference versus female controls (p > 0.05). 24-h urinary K^+ ^excretion per 100 g of body weight in NaVPA-male rats was significantly higher than in control males (p = 0.025; Table 1). No gender-related differences in this index were found in NaVPA-rats. This index in NaVPA-female rats did not show any statistically significant difference as compared with female controls (p > 0.05).

The K^+^/Cl^- ^ratio (Table 2) in NaVPA-males and NaVPA-females was significantly lower as compared to control males (p < 0.0005) and control females (p < 0.0005), without any statistically significant gender-related differences.

**Table 2:** 24-h urinary chloride (Cl^-^) excretion data and K^+^/Cl^- ^ratio in male and female control and NaVPA-rat groups (mean ± SD)

Rat groups	n	24-h Cl^- ^level (mmol/l)	24-h Cl^- ^excretion (mmol/24 h)	24-h Cl^- ^excretion per 100 g body weight (mmol/100 g)	Cl^-^/creatinine ratio	K^+^/Cl^- ^ratio
Control rats						
females	14	104 ± 51	0.886 ± 0.40	0.371 ± 0.15	12.6 ± 6.9	1.30 ± 0.44
males	14	168 ± 128	1.164 ± 0.70	0.420 ± 0.26	14.2 ± 8.8	1.15 ± 0.54
NaVPA-rats						
females	13	180 ± 60^•^	1.997 ± 0.52^• ^*	0.833 ± 0.22^• ^*	24.6 ± 4.7^• ^*	0.49 ± 0.12^•^
males	13	275 ± 184^•^	3.396 ± 1.21^• ^*	1.208 ± 0.53^• ^*	34.6 ± 16.5^• ^*	0.45 ± 0.22^•^

^• ^– Statistically significant difference, as compared to control group (p < 0.05).

* – Statistically significant difference versus the other gender (p < 0.05).

### Cl^- ^excretion in control and NaVPA-rats

No significant gender differences of 24-h urine, Cl^- ^excretion and Cl^- ^excretion per 100 g of body weight and Cl^-^/creatinine ratio were determined in control rats, either (p > 0.05; Table 2).

Compared to control, 24-h urinary Cl^- ^levels were significantly (p < 0.05) higher in both genders of experimental animals. 24-h urinary Cl^- ^excretion, 24-h urinary Cl^- ^excretion per 100 g of body weight and the Cl^-^/creatinine ratio in both sexes of NaVPA-rats were significantly higher than in gender-matched controls (p < 0.001). Chloride excretion (24-h urinary Cl^- ^excretion, 24-h urinary Cl^- ^excretion per 100 g of body weight and the Cl^-^/creatinine ratio) was found significantly higher in NaVPA-male than in NaVPA-female rats (p < 0.05; Table 2). 24-h creatinine excretion in NaVPA-male rats (0.110 ± 0.025 mmol) was significantly higher than in NaVPA-female rats (0.082 ± 0.017 mmol; p < 0.005). No gender-related difference in 24-h creatinine excretion between control male and female rats was determined (p > 0.05).

The 24-h urine pH showed no statistically significant difference between control females (6.57 ± 0.2) and control males (6.53 ± 0.2; p > 0.05). The 24-h urine pH in NaVPA-males (6.42 ± 0.3) and NaVPA-females (6.43 ± 0.3) showed no statistically significant differences from controls and between the genders (p > 0.05). In NaVPA-rats, only the correlation between Cl^- ^excretion and 24-h urine pH was significant in NaVPA-males (r = -0.73; p < 0.05, Figure 3). The correlation coefficient of 24-h urinary Cl^- ^excretion and urine pH statistically significantly differed (p < 0.02) between control males (r = 0.03) and NaVPA-males (r = -0.73), also between NaVPA-females and NaVPA-males (r = 0.02, r = -0.73, p < 0.05), without any statistically significant differences in control females and males (p > 0.05).

The correlation between 24-h urinary K^+ ^and Cl^- ^excretion and between respective electrolyte and 24-h urine pH in control male and female rats was not statistically significant (p > 0.05). The correlation coefficient of 24-h urinary K^+ ^excretion and 24-h urinary Cl^- ^excretion statistically significantly differed (p = 0.03) only between control males (r = -0.28) and control females (r = 0.49).

## Discussion

Cl^- ^and K^+ ^fluxes play a crucial role in synaptic inhibition, cell pH regulation, as well as in cell volume control and tissue susceptibility to seizures [16,17]. A recent study showed that NaVPA, alongside the diuretic effect, enhances sodium and Cl^- ^excretion with urine [3]. Acute and subacute administration of valproic acid has been shown to exert a moderate diuretic effect on rats [9,18,19].

The study data showed that intragastric 300 mg/kg NaVPA significantly increased 24-h K^+ ^excretion in the urine of NaVPA-male rats. 24-h K^+ ^excretion in NaVPA-male rats was significantly higher than in control males and NaVPA-female rats. 24-h urinary K^+ ^excretion per 100 g of body weight in NaVPA-male rats was significantly higher than in control male rats.

The enhanced excretion of K^+ ^and Cl^- ^with urine and the related gender differences of NaVPA effect could be important for elucidating the pathophysiological phenomena related to NaVPA pharmacology. GABA functions appear to be triggered by GABA binding to its ionotropic receptors, which are ligand-gated Cl^- ^channels [4,5]. The Cl^- ^channel of the GABA(A) receptor is activated by valproic acid in brain cells [7]. GABA was also found to activate K^+ ^conductance in the central nervous system [20], it is involved in a wide variety of physiological functions in tissues and organs outside the brain [12,21]. Activation of the GABA(A) receptor leads to a stimulation of Na^+^-K^+^-2Cl^- ^cotransporter in brain cells, and this results in a loss of intracellular Cl^- ^[22] or in an upregulation of KCC which is important in maintaining the low intracellular Cl^- ^level [23].

Studies demonstrating K^+^-Cl^- ^cotransport in rabbit proximal tubules have shown a coupled K^+^-Cl^- ^movement from the cell to peritubular fluid [24]. In addition, several modes of coupled K^+ ^and Cl^- ^movement have been shown in K^+ ^excretion by renal tubules: directly coupled K^+^-Cl^- ^cotransport, parallel K^+ ^and Cl^- ^conductance, parallel K^+^/H^+ ^and Cl^-^/HCO3^- ^exchangers and Na^+^-K^+^-2Cl^- ^cotransport [25]. In modeling Cl^- ^transport in the rat proximal tubule, Weinstein found that Cl^- ^effluxes from the cell predominantly via K^+^-Cl^- ^cotransport [13].

The presence and physiological significance of GABA or GABA(A) receptors in nonneural tissue is less clear [26]. The effect of pharmacological manipulation of GABAergic transmission on KCC activity in the kidney remains to be clarified. GABA immunoreactivity in the rat kidney was predominantly confined to renal tubules, including the ascending parts of the distal tubules, and the loop of Henle, the collecting tubules and ducts, and the connective parts of the convoluted tubules. The high K^+ ^concentration evoked an efflux of endogenous GABA from rat kidney slices. GABA released from renal tubular epithelium and transported with urine might be involved in the modulation of K^+ ^transport in the urinary tract cells [27]. However, our findings allow to hypothesize that GABA(A) subunits may play a role in basolateral membrane Cl^- ^transport. These findings support the suggestion of other investigators, that subunits of the ligand-gated Cl^- ^channel superfamily may be involved in renal Cl^- ^excretion [6].

NaVPA exerts a gender-related effect on urinary Cl^- ^excretion: 24-h urinary Cl^- ^excretion was significantly higher in rats of both sexes, but Cl^- ^excretion was significantly higher in male than in female rats. In NaVPA-male rats, the negative correlation between Cl^- ^excretion and 24-diuresis and between Cl^- ^excretion and urine pH was significant, but such correlations were not characteristic of control male or NaVPA-female rats. The K^+^/Cl^- ^ratio in NaVPA-males and NaVPA-females was significantly lower as compared to control males and control females, without gender-related differences. There are no data that could contribute to elucidating the mechanisms of NaVPA-induced enhanced urinary Cl^- ^excretion. It is known that the alkaline extracellular pH increases the GABA(A) channel opening frequency and decreases the duration of the long-closed state in rat hypothalamus [28]. There are no data that NaVPA influences tubular intracellular and extracellular pH. Gender-related differences in intracellular Cl^- ^concentration and Cl^- ^transport in smooth muscle cells of male and female rats have been reported [9]. Sexual dimorphism in the expression of KCC, GABA receptors and in GABA release in the nervous system of rats is a known fact [29-35].

Tissue distribution studies with radiolabeled NaVPA in rodents have shown that NaVPA distributed mainly in the extracellular space; high levels of radiolabeled NaVPA were found in the liver and kidney [36]. We failed to find data on gender-related differences of NaVPA pharmacokinetics, pharmacodynamics, or gender differences of NaVPA metabolism in rats. The NaVPA-induced K^+ ^and Cl^- ^excretion enhancement may be related to the upregulated renal hemodynamics, as the NaVPA doses applied are known to reduce arterial blood pressure in rats: NaVPA provoked a prolonged cardiovascular depression which was very similar to that observed with high i.v. doses of GABA [18,37]. The mechanism of the cardio depressive effect of NaVPA is not clear; it seems not to involve interference with peripheral vascular noradrenergic activity or arterial baroreflex control [38]. Furthermore, Cl^- ^is an important factor of renal vascular tone or renal hemodynamics regulation. Renal arterial infusion of Cl^- ^acts as a direct vasoconstrictor [39]; retrograde injection of Cl^-^-containing solution into the distal tubule elicits a decrease in a single nephron glomerular filtration rate, whereas injection of sodium ion containing solution without Cl^- ^does not [40]. In oncotically perfused rat kidneys, in the presence of either high or low Cl^- ^with sodium kept constant, low Cl^- ^resulted in a higher glomerular filtration rate than with high chloride [41]. It is not likely that the NaVPA-induced gender-related K^+ ^and Cl^- ^excretion is linked to Na^+^-K^+^-2Cl^- ^cotransporter inhibition, because the Na^+^-K^+^-2Cl^- ^cotransporter inhibitor furosemide exerts an opposite gender-related chloriduretic effect in rats [42].

## Conclusion

NaVPA, alongside the diuretic effect, enhances K^+ ^and Cl^- ^excretion with urine in rats of both genders. The mechanism of this different gender-dependent effect is not yet clear. The experimental observations reported above may have potentially important pharmacological implications. Elucidation of NaVPA-induced mechanisms of enhanced K^+ ^and Cl^- ^excretion could be of value while explaining the pharmacological basis of NaVPA action. Thus, further studies of the mechanisms of NaVPA effects on K^+ ^and Cl^- ^transport in cells could be important.

## Abbreviations

NaVPA = sodium valproate

Cl^- ^= chloride

K^+ ^= potassium

KCC = K-Cl co-transporter

## Authors' contributions

All coauthors took part in conceiving, designing and coordinating the study; Jakutiene E and Grikiniene J performed the experiments; Grikiniene J and Vaitkevicius A analysed the data; Jakutiene E, Stakisaitis D wrote the paper. All authors were involved in drafting, revising and approving the manuscript.
